# Parental Protectiveness Mediates the Association between Parent-Perceived Child Self-Efficacy and Health Outcomes in Pediatric Functional Abdominal Pain Disorder

**DOI:** 10.3390/children3030015

**Published:** 2016-09-19

**Authors:** Melissa M. DuPen, Miranda A. L. van Tilburg, Shelby L. Langer, Tasha B. Murphy, Joan M. Romano, Rona L. Levy

**Affiliations:** 1 School of Social Work, University of Washington, Seattle, WA 98105, USA; tbmurphy@uw.edu (T.B.M.); rlevy@uw.edu (R.L.L.); 2 Center for Functional GI and Motility Disorders, University of North Carolina, Chapel Hill, NC 27599, USA; tilburg@med.unc.edu; 3 College of Nursing and Health Innovation, Arizona State University, Phoenix, AZ 85004, USA; Shelby.Langer@asu.edu; 4 Psychiatry and Behavioral Sciences, University of Washington, Seattle, WA 98105, USA; jromano@uw.edu

**Keywords:** Functional Abdominal Pain Disorder, self-efficacy, somatic symptoms, disability, parenting, protectiveness, children, pain, coping

## Abstract

Previous studies have shown that parental protectiveness is associated with increased pain and disability in Functional Abdominal Pain Disorder (FAPD) but the role that perceived child self-efficacy may play remains unclear. One reason why parents may react protectively towards their child’s pain is that they perceive their child to be unable to cope or function normally while in pain (perceived low self-efficacy). This study sought to examine (a) the association between parent-perceived child pain self-efficacy and child health outcomes (symptom severity and disability); and (b) the role of parental protectiveness as a mediator of this association. Participants were 316 parents of children aged 7–12 years with FAPD. Parents completed measures of perceived child self-efficacy when in pain, their own protective responses to their child’s pain, child gastrointestinal (GI) symptom severity, and child functional disability. Parent-perceived child self-efficacy was inversely associated with parent-reported child GI symptom severity and disability, and parental protectiveness mediated these associations. These results suggest that parents who perceive their child to have low self-efficacy to cope with pain respond more protectively when they believe he/she is in pain, and this, in turn, is associated with higher levels of GI symptoms and disability in their child. This finding suggests that directly addressing parent beliefs about their child’s ability to manage pain should be included as a component of FAPD, and potentially other child treatment interventions.

## 1. Introduction

One of the most common recurrent pain complaints of childhood is abdominal pain, which affects approximately 13.5% of children worldwide [1]. Many children with persistent abdominal pain meet criteria for Functional Abdominal Pain Disorder (FAPD), defined as episodic or continuous abdominal pain without evidence of physiological etiology [2]. FAPD accounts for approximately 3% of visits to pediatricians [3], more than 50% of the referrals to gastroenterology clinics [4], and is the main reason for gastrointestinal (GI) emergency room visits among children [5]. Children with FAPD not only experience frequent abdominal pain that interferes with normal activities, but also have higher rates of disability and other somatic complaints. 

The cognitions, psychological state, and pain behaviors of parents have all been shown to be related to pain behaviors and functioning in children. Parental protective and solicitous responses to child pain behavior (parental protectiveness) are responses that tend to buffer the child from normal demands/activities or provide additional attention or rewards in response to child pain behaviors (e.g., giving the child special treats and/or keeping them home from school when in pain). Previous research has shown that parental protectiveness is associated with increased pain, disability, and somatic complaints in children with FAPD [6]. One logical hypothesis as to why parents react to their child’s pain protectively is that they may believe that their child is unable to deal with and function normally while having pain (perceived low pain self-efficacy).

Self-efficacy was first conceptualized by Bandura as part of the Social Cognitive Theory [7,8,9]. Self-efficacy in a general context is a person’s belief in his/her ability to perform a task or obtain a goal. Rosenstock [10], for example, suggested incorporating self-efficacy into the Health Belief Model, a model used to better understand individual health-related behaviors such as keeping preventive health appointments, adherence to treatment, and general confidence in medical care [11].

**Figure 1:**
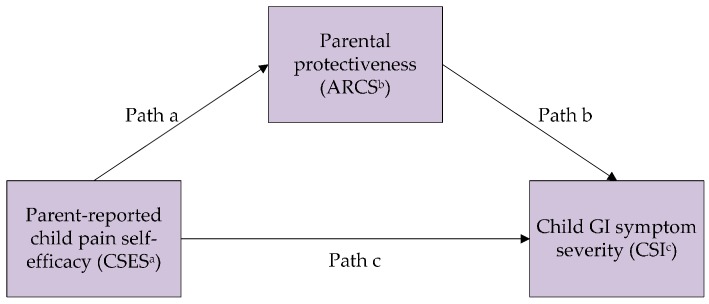
Mediation model 1—Parent-perceived child pain self-efficacy, parental protectiveness, and parent-reported child gastrointestinal (GI) symptom severity. ^a^ CSES: Child Self-Efficacy Scale; ^b^ ARCS: Adult Responses to Children’s Symptoms—Protectiveness subscale; ^c^ CSI: Children’s Somatization Inventory—GI subscale.

**Figure 2:**
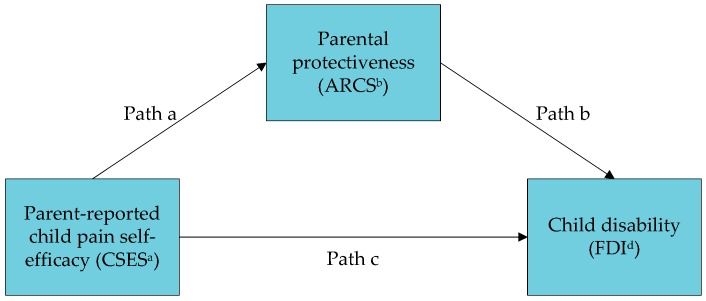
Mediation model 2—Parent-perceived child pain self-efficacy, parental protectiveness, and parent-reported child disability. ^a^ CSES: Child Self-Efficacy Scale; ^b^ ARCS: Adult Responses to Children’s Symptoms—Protectiveness subscale; ^d^ FDI: Functional Disability Inventory.

Relatedly, “pain self-efficacy” is a term defined as one’s certainty or belief in one’s ability to function normally when experiencing pain [12,13]. Studies have found that greater pain self-efficacy was associated with lower levels of disability in children with chronic headaches [14], and with lower levels of somatic symptoms in children with chronic pain [13]. However, the mechanisms by which pain self-efficacy might be related to symptoms and disability are unclear. Parental protectiveness is a plausible mechanism by which perceived self-efficacy may be related to health outcomes. Based on these considerations, this study sought to examine (a) the association between parent-perceived child pain self-efficacy and parent-reported child health outcomes (symptom severity and disability); and (b) the role of parental protectiveness as a mediator of these associations (Figure 1 and Figure 2). 

## 2. Materials and Methods

### 2.1. Participants

Participants were 316 parents or caregivers of children enrolled in a large multi-site cognitive-behavioral intervention study for parents of children with FAPD [15]. Families were recruited from four pediatric gastroenterology clinics located in Seattle, WA, USA; Tacoma, WA, USA; Chapel Hill, NC, USA; and Bend, OR, USA. Children and parents were included in the study if the child was aged 7 to 12 years, lived with the parent or caregiver for at least the past three months, had a gastroenterologist-documented diagnosis of FAPD based on ROME III criteria [16], had abdominal pain at least once a week over the past two months, and spoke English. Families were excluded if the child had positive physical or laboratory findings that would explain the abdominal pain, any chronic disease, major surgery within the past year, or a developmental disability requiring full-time special education or that impaired their ability to participate. 

The institutional review boards at each clinic location approved the study. Recruiters from each location screened potential participants based on the inclusion and exclusion criteria listed above and obtained verbal or written consent. 352 participants consented to be in the study; of those 316 completed baseline assessments and were randomized, and 36 did not complete baseline assessments and thus were not randomized (28 did not respond, five said they were too busy, and three did not complete for other reasons). Participants were randomly assigned to either a social learning and cognitive-behavioral therapy condition administered by phone or in-person; or an education and support condition administered by phone. Topics included teaching parents to model and reinforce wellness behaviors, decrease reinforcement of illness behaviors, reduce negative cognitions regarding FAPD; or education about the GI system, nutritional guidelines, and food safety.

### 2.2. Measures

All measures were completed by parents and collected online or by mail (over 90% completed online) at baseline, prior to randomization into the intervention component of the study. Measures are described in detail below.

#### 2.2.1 Child Self-Efficacy Scale (parent-report) (CSES; [13])

The CSES assesses parent beliefs about their child’s ability to function normally when in pain. The measure contains seven items rated on a 1–5 scale and was reverse-scored for ease of interpretation such that higher scores equal higher parent-perceived child pain self-efficacy. Example items are “How sure are you that your child can do well in school when in pain?” and “How sure are you that your child can do house chores when in pain?” This measure has demonstrated excellent reliability and validity for both parent and child-report versions and the developers reported an internal consistency (Cronbach’s coefficient alpha) of 0.89 [13]. The internal consistency for this sample was 0.93. 

#### 2.2.2. Adult Responses to Children’s Symptoms Scale (ARCS; [17,18,19])

The ARCS assesses parent responses to children’s pain. We focused on the protectiveness subscale which has been recently revised to contain 13 items [19] rated on a 0–4 scale (higher scores indicate more protective and solicitous responses to pain behavior) and has been validated in a large sample of mothers [17]. Example items are “When your child has abdominal pain how often do you pay more attention to your child than usual?” and “When your child has abdominal pain how often do you keep your child inside the house?” Developers of the measure reported an internal consistency of 0.86. Internal consistency in the current sample was also found to be 0.86.

#### 2.2.3. Children’s Somatization Inventory (parent-report) (CSI; [20])

The CSI measures parent-report of their child’s symptom severity during the past two weeks. We used the seven-item GI subscale (nausea or upset stomach; constipation; loose bowel movements or diarrhea; stomachaches; vomiting; feeling bloated or gassy, and food making child sick) which has been shown to be a valid and reliable measure of GI symptoms [21]. Items are rated on a 0–4 scale where higher scores indicate greater symptom severity. Internal consistency for a large sample of children with unexplained abdominal pain was 0.75 [22]; it was 0.68 for the current sample. 

#### 2.2.4. Functional Disability Inventory (parent-report) (FDI; [23])

The FDI asks parents to rate their child’s difficulty doing regular activities such as “walking up stairs” and “being at school all day”, over the past few days. This measure includes 15 items rated on a 0–4 scale, where higher scores indicate greater disability. Developers of the measure reported an internal consistency of 0.94 in a sample of mothers [23]. Internal consistency for this sample was also found to be 0.94.

### 2.3. Data Analysis

Analyses were conducted using IBM Statistical Package for the Social Sciences (version 19, Armonk, NY, USA). Descriptive analyses of demographic data for parents and children were conducted, followed by Pearson correlations to examine bivariate associations within parent-report measures. Two separate mediation analyses were then performed using the Hayes’ PROCESS macro [24] to predict child GI symptom severity (Figure 1) and child disability (Figure 2). The Hayes’ PROCESS macro is a regression-based path analytic framework [24] that calculates the regression of the outcome on the predictor, the regression of the mediator on the predictor, and the regression of the outcome on both the predictor and mediator. Bootstrap methods were used to test for an indirect effect and to compute bias-corrected confidence intervals for this effect, with 10,000 resamples. In both cases, parent-perceived child self-efficacy was treated as the predictor and parental protectiveness as the mediator.

## 3. Results

### 3.1. Descriptive Statistics

Demographic characteristics of the parents in the study were: mean (standard deviation, SD) age of 39.9 (7.4) years; 94.9% female; 84.8% White; 7.0% Hispanic or Latino; 47.2% with a 4-year college degree or higher; 57.3% employed at least part-time; and 79.4% married or cohabitating with a partner. Demographic characteristics of the children were: mean (SD) age of 9.4 (1.6) years; 64.6% female; 81.6% White; and 9.8% Hispanic or Latino. 

Table 1 presents a correlation matrix, as well as descriptive statistics for study variables. Parent-perceived child self-efficacy was inversely correlated with parental protectiveness (*r* = −0.55, *p* < 0.01), parent-reported child GI symptom severity (*r* = −0.25, *p* < 0.01), and parent-reported child disability (*r* = −0.43, *p* < 0.01). These inverse relationships indicate that lower scores on the CSES (i.e., lower self-efficacy) were correlated with higher levels of parental protectiveness, child GI symptom severity, and child disability. Parental protectiveness was positively correlated with child GI symptom severity (*r* = 0.25, *p* < 0.01) and child disability (*r* = 0.39, *p* < 0.01); and child GI symptom severity was positively correlated with child disability (*r* = 0.46; *p* < 0.01). 

### 3.2. Mediation Analyses

Table 2 shows the results of the mediation analysis for Model 1 where parent-perceived child self-efficacy was the predictor, parental protectiveness was the mediator, and parent-reported child GI symptom severity was the outcome. Parent-perceived child self-efficacy was inversely associated with both child GI symptom severity (Path c, estimate (standard error—SE) = −0.19 (0.04), *p* < 0.001) and parental protectiveness (Path a, estimate (SE) = −0.39 (0.03), *p* < 0.001). Parental protectiveness was positively associated with child GI symptom severity (Path b, estimate (SE) = 0.18 (0.07), *p* = 0.011). Our mediation hypothesis was supported by a significant indirect effect (Path a × b, estimate (SE) = −0.07 (0.03), 95% CI with 10,000 resamples = −0.13, −0.01). The ratio of the indirect effect to the total effect, or the proportion of the total effects mediated for Model 1 was 37%. 

Table 3 shows the results of the mediation analysis for Model 2 where parent-perceived child self-efficacy was the predictor, parental protectiveness was the mediator, and parent-reported child disability was the outcome. This model showed similar results. Parent-perceived child self-efficacy was inversely associated with both child disability (Path c, estimate (SE) = −0.34 (0.04), *p* < 0.001) and parental protectiveness (Path a, estimate (SE) = −0.39 (0.03), *p* < 0.001). Parental protectiveness was positively associated with child disability (Path b, estimate (SE) = 0.24 (0.07), *p* < 0.001). Our mediation hypothesis was also supported here by a significant indirect effect (Path a × b, estimate (SE) = −0.10 (0.03), 95% CI with 10,000 resamples = −0.16, −0.04). The ratio of the indirect effect to the total effect for Model 2 was 28%.

## 4. Discussion

The goal of this study was to examine whether parents respond with greater protectiveness if they perceive their child to have lower pain self-efficacy and whether this protectiveness is associated with more child GI symptoms and greater child disability. Results suggest support for this hypothesis. Parents who perceived their child’s self-efficacy as low responded more protectively to their child when he/she was in pain and this was associated with higher levels of GI symptoms and disability in their child. 

The role of self-efficacy in pediatric pain has been previously described with studies suggesting that lower self-efficacy is associated with more symptoms and greater disability [13,14]. Parent protectiveness has also been positively associated with symptoms and disability [6,25,26]. In the current study we not only replicated these findings, but our analyses further showed that parental perception of child pain self-efficacy may be driving parental protectiveness. Our findings suggest several areas for intervention. For example, cognitive interventions targeting parental perceptions of their child’s pain may be effective in reducing child’s symptoms and disability. In our own treatment trial, we have shown that reducing parental perception of the threat of child’s pain mediated treatment outcomes [27] which lends support for parental intervention. Alternatively, as we do not know if parents wrongly assign low self-efficacy in their children or even if their judgment is correct, improving child self-efficacy may be a helpful target for treatment via self-management, relaxation training, or other methods to reduce the impact of FAPD.

Limitations of this study should be noted. All measures used in this study were parent-report, although developers of the self-efficacy measure used in this study found that parent and child-reports were significantly correlated with each other [13]. Nonetheless, the fact that parents were the only source of data increases the possibility of introducing single source bias, a type of common method variance, into the findings, potentially limiting their validity. Given that this is an inherent limitation of the study methods, it should be kept in mind when interpreting the findings. While evidence for mediation was found, the proportion of total effects accounted for was 28% and 37%, indicating that other factors besides parental protectiveness may play an important role in mediating the relationship between perceived child self-efficacy and outcomes. Further studies using multiple data sources and expanded mediational models are needed to address these limitations. It should also be noted that ninety-five percent of the parents were female, which could limit the generalizability of the findings. Studies including the effect of fathers’ cognitions and behaviors are needed. The sample may also be biased by including parents who were willing to participate in a treatment trial for FAPD and therefore may not represent the entire population of FAPD children and their parents. Another study limitation is our cross-sectional design, which makes it difficult to determine cause and effect. We do not know if parental perceptions of child self-efficacy lead to parental protectiveness, if parental protectiveness affects parental perceptions of efficacy, or if there is a bidirectional relationship between the two. In addition, parents who perceive their child as more disabled or symptomatic may also respond more protectively and perceive their child as less effective in self-managing their pain. Longitudinal studies and treatment studies addressing parent cognitions and/or improving child self-efficacy are needed to address these questions of directionality.

## 5. Conclusions

Parents may respond protectively to their child (e.g., by keeping them home from school when in pain) if they perceive their child to be unable to deal with pain and function normally when in pain, and this is associated with higher child GI symptoms and disability. These findings suggest that directly addressing parent beliefs about their child’s ability to manage his/her pain should be included as a component of FAPD, and potentially other child treatment interventions.

## Figures and Tables

**Table 1:** Correlations among study variables and descriptive statistics (*n* = 316).

	Protect (ARCS)	GI Symptoms (CSI-GI)	Disability (FDI)	M (SD)	Scale
CSES ^a^	−0.55 **	−0.25 **	−0.43 **	3.30 (0.91)	1–5
ARCS ^b^		0.25 **	0.39 **	1.17 (0.65)	0–4
CSI-GI ^c^			0.46 **	1.42 (0.68)	0–4
FDI ^d^				0.70 (0.73)	0–4

** *p* < 0.01; ^a^ CSES: Child Self-Efficacy Scale; ^b^ ARCS: Adult Responses to Children’s Symptoms—Protectiveness subscale; ^c^ CSI: Children’s Somatization Inventory—GI subscale; ^d^ FDI: Functional Disability Inventory. GI: gastrointestinal.

**Table 2:** Results of mediation analysis for Model 1 (parent-reported child GI symptom severity).

	Estimate (SE)	*p*	95% CI
Path c (effect of predictor on outcome)	−0.19 (0.04)	<0.001	
Path a (effect of predictor on mediator)	−0.39 (0.03)	<0.001	
Path b (effect of mediator on outcome)	0.18 (0.07)	0.011	
Indirect effect: Path a × b	−0.07 (0.03)		−0.13, −0.01
Direct effect: Path c’	−0.12 (0.05)	0.016	
Ratio of indirect effect to total effect	37%		

SE: standard error; CI: confidence interval.

**Table 3:** Results of mediation analysis for Model 2 (parent-reported child disability).

	Estimate (SE)	*p*	95% CI
Path c (effect of predictor on outcome)	−0.34 (0.04)	<0.001	
Path a (effect of predictor on mediator)	−0.39 (0.03)	<0.001	
Path b (effect of mediator on outcome)	0.24 (0.07)	<0.001	
Indirect effect: Path a × b	−0.10 (0.03)		−0.16, −0.04
Direct effect: Path c’	−0.25 (0.05)	<0.001	
Ratio of indirect effect to total effect	28%		
